# Subscribers’ Perspectives and Satisfaction with the MoreGoodDays Supportive Text Messaging Program and the Impact of the Program on Self-Rated Clinical Measures

**DOI:** 10.3390/jcm13020580

**Published:** 2024-01-19

**Authors:** Belinda Agyapong, Reham Shalaby, Ejemai Eboreime, Katherine Hay, Rachal Pattison, Mark Korthuis, Yifeng Wei, Vincent Israel Opoku Agyapong

**Affiliations:** 1 Department of Psychiatry, Faculty of Medicine and Dentistry, University of Alberta, Edmonton, AB T6G 2B7, Canada; 2 Department of Psychiatry, Faculty of Medicine, Dalhousie University, Halifax, NS B3H 2E2, Canada; 3 Kickstand, Edmonton, AB T5K 2J5, Canada; 4 Glenrose Rehabilitation Hospital Foundation, Edmonton, AB T5G 0B7, Canada

**Keywords:** MoreGoodDays, satisfaction, young adults, cognitive behavioural therapy, text messages

## Abstract

**Background:** Young adults (18 to 30 years of age) are confronted with numerous challenges, such as academic stressors and peer pressure. The MoreGoodDays program was co-designed with young adults to alleviate psychological issues, improve their mental well-being and provide support for young adults in Alberta during the COVID-19 pandemic and beyond. **Objective:** The current study aimed to explore subscribers’ perspectives and satisfaction with the MoreGoodDays supportive text messaging program and the impact of the program on self-rated clinical measures. **Methods:** Subscribers of the MoreGoodDays program were invited via a link delivered in a text message to complete online evaluation surveys at six weeks, three months and six months. Program perception and satisfaction questions were adapted from those used to evaluate related programs. Anxiety, depression and PTSD symptoms were respectively assessed using the Generalized Anxiety Disorder-7 scale, the Patient Health Questionnaire-9 scale and the PTSD Civilian Checklist 5, and resilience levels were assessed using the Brief Resilience Scale (BRS). Data were analyzed with SPSS version 26 for Windows utilizing descriptive and inferential statistics. **Results:** There was a total of 168 respondents across the three follow-up time points (six weeks, three months and six months). The overall mean satisfaction with the MoreGoodDays program was 8.74 (SD = 1.4). A total of 116 (69.1%) respondents agreed or strongly agreed that MoreGoodDays messages helped them cope with stress, and 118 (70.3) agreed the messages helped them cope with loneliness. Similarly, 130 (77.3%) respondents agreed that MoreGoodDays messages made them feel connected to a support system, and 135 (80.4) indicated the program helped to improve their overall mental well-being. In relation to clinical outcomes, the ANOVA test showed no significant differences in mean scores for the PHQ-9, GAD-7 and PCL-C scales and the BRS from baseline to the three follow-up time points. In addition, there was no statistically significant difference in the prevalence of likely GAD, likely MDD, likely PTSD and low resilience at baseline and at six weeks. **Conclusions:** Notwithstanding the lack of statistically significant clinical improvement in subscribers of the MoreGoodDays program, the high program satisfaction suggests that subscribers accepted the technology-based intervention co-created with young adults, and this offers a vital tool to complement existing programs.

## 1. Introduction

Young adults (18 to 30 years) are constantly faced with numerous challenges, including academic stress, peer pressure and financial stressors. Research prior to the COVID-19 pandemic indicates that this group experienced high levels of stress, anxiety and depression [1,2], which were further exacerbated by the pandemic [3]. Anxiety and depression are frequently comorbid and result in impairment in young adults [4,5,6]. The adverse impact of these psychological issues interferes with interpersonal relationships, social interactions and academic achievement and is a risk factor for suicide and other psychiatric problems [7,8,9]. A survey conducted in China among college students reported that the prevalence of anxiety and depressive disorders was 11.0% and 21.1%, respectively [10]. Another study [11] in China reported a significantly higher prevalence of generalized anxiety disorder (GAD) and major depressive disorder (MDD) in individuals less than 35 years in comparison with those aged 35 years or older. Similarly, a recent study among adolescents and young adults in Canada reported a relatively high prevalence of likely MDD (56.0%) and likely GAD (46.6%) [3]. Another Canadian study collaborated on this finding, reporting comparatively high mean scores on self-rated scales for stress, anxiety and depression among participants 25 years or less [12]. In yet another study that sought to identify factors associated with depression, anxiety and PTSD symptomatology in U.S. young adults (18–30 years) during the COVID-19 pandemic, it was reported that 43.3% of the sample had high levels of depression (PHQ-8 scores ≥ 10), 45.4% had high anxiety scores (GAD-7 scores ≥ 10), and 31.8% had high levels of PTSD symptoms (PCL-C scores ≥ 45) [13]. In this study, it was reported that resilience was associated with low levels of depression and anxiety symptoms but not PTSD.

With the exponential increase in psychological issues among young adults, it is necessary to adopt interventions likely to be embraced and accepted by this group. The use of technology, in particular, mobile phones for e-communication such as instant messaging and social media, is more prominent and utilized among young adults than in older people, making text messaging apps for the delivery of cognitive behavioral therapy (CBT)-based interventions a suitable option [14,15]. CBT-based interventions have been implemented in several healthcare segments with good outcomes for different psychiatric issues, including depression, anxiety and personality disorders [16,17]. An advantage of using e-communication mental health tools is the ease of accessibility for young adults who generally envisage using technology as natural, suitable and desirable, aligning with their need for autonomy and confidentiality [14]. Mental health management and assessment mobile phone application, the “mobiletype” program, for instance, has been used among the youth to monitor mood, stress and coping strategies, among other things [18]. Both natural such as wildfires and manmade disasters such as wars can have devastating consequences on the psychological well-being of impacted residents, particularly the youth. The Russian invasion of Ukraine, for example, has had an adverse effect on the mental health of Ukrainian adolescents and young adults and caused considerable financial, social and political instability worldwide [19]. During such a crisis, supportive text messages can be used to support, instill hope and improve the mental well-being of this cohort. The development of modern telecommunication techniques became particularly useful during the initial stages of the COVID-19 pandemic [20]. In Alberta, the MoreGoodDays program was developed during the COVID-19 pandemic to address psychological problems in young adults. 

## 2. MoreGoodDays Program

The MoreGoodDays program was co-developed with young adults to alleviate anxiety, depression and PTSD symptoms, increase resilience and improve the general mental well-being of their peers. The MoreGoodDays is modeled on the award-winning Text4Mood program [21], which was launched in Northern Alberta to address long waitlist and geographical barriers to the receipt of counseling services, and the Text4Hope program, which was launched during the COVID-19 pandemic to mitigate symptoms of stress, anxiety and depression among the general public in Alberta [22]. Participants could subscribe by texting “MoreGoodDays” to a designated short code number Data were collected between 28 January 2021 and 17 July 2022. Participants also have the option to unsubscribe at any time by texting “stop”. MoreGoodDays was advertised by Kickstand in Alberta [23], and the program messages are delivered through an online application, the ResilienceNHope [24,25]. ResilienceNHope is an e-mental health application that delivers one-way (non-interactive) CBT-based supportive messages via mobile text or email to offer psychological support to help address part of the mental health treatment gap for individuals and communities globally. The MoreGoodDays program offers one year of free non-personalized and non-interactive daily text messaging service to the youth in Alberta [23]. All subscribers receive the same messages throughout the duration of their subscription to the program, and the message received each day depends on the number of days that have lapsed from subscription. Individuals who subscribe to the program on the same day receive the same message each day throughout their subscription. Samples of text messages received by those who subscribe to the program are as follows:

- YOU ARE AMAZING! Your light deserves to shine bright! Be gentle, kind and compassionate to yourself. Do what makes you happy and don’t be afraid to let your light shine! YOU GOT THIS!
- Remember: It is okay to outgrow people, places and things. “If you are outgrowing who you’ve been, you are on the right schedule, keep evolving”—Lalah Delia
- Figuring out who you are takes time and a lot of effort. Even when you’re old you’ll be finding out new things about yourself, so it’s ok if you don’t know right now. You’ll figure it out eventually.

A significant difference between the MoreGoodDays program and Text4Hope and Text4Mood programs is that unlike the Text4Hope and Text4Mood programs, for which all messages were developed by mental health professionals in collaboration with service user representatives based on CBT principles, the MoreGoodDays program messages were written entirely by young adults based on the Text4Hope conceptual framework as educational resources and subjected to a safety review by a clinical team. The messages were not necessarily CBT-based. Young adult subscribers of Text4Hope reported a significant reduction in the prevalence of moderate to high stress, likely GAD, likely MDD and suicidal ideation after six weeks of receiving the daily messages [22]. Subscribers to the Text4Hope and Text4Mood programs have high satisfaction with the services received [21,26]. In this regard, the current study aims to assess subscribers’ perspectives and satisfaction with the MoreGoodDays supportive text messaging program, as well as the impact of the program on self-rated clinical measures. 

Our specific objectives are as follows:

- To assess MoreGoodDays subscribers’ perceptions of the daily messages.
- To assess subscribers’ satisfaction with the MoreGoodDays supportive text messaging program.
- To assess MoreGoodDays subscribers’ receptivity to having various technology-enabled services incorporated into healthcare delivery.
- To evaluate the impact of the daily supportive text messages on the severity and prevalence of likely GAD, likely MDD, low resilience and likely PTSD among subscribers of the MoreGoodDays program.

Given that the MoreGoodDays program was modeled on the Text4Hope and Text4Mood [22] programs, which reported high subscriber satisfaction and clinical effectiveness in reducing psychological symptoms, we hypothesize that participants of the MoreGoodDays program will express high satisfaction, positive perceptions and regard for other technology-based healthcare tools. We also hypothesize that there will be statistically significant improvements in all measures of anxiety, depression, PTSD and resilience from baseline to the follow-up time points. 

The outcome of this evaluation will be important in evaluating if this population-based service can be an add-on aid in future service planning for young adults. 

## 3. Methods

### 3.1. Study Setting and Design

The study was conducted in Alberta, Canada, with an estimated population of 4,601,314, with 11.9% being youth [27]. This study employed a cross-sectional and a longitudinal cross-sectional or sequential design to examine perceptions, satisfaction and clinical outcomes among MoreGoodDays participants who completed the online baseline surveys at six weeks, three months and six months. Data collection took place between 28 January 2021 and 17 July 2022. 

### 3.2. Ethics Statement and Consent

The research was performed according to the Declaration of Helsinki. Study was approved by the University of Alberta Health Research Ethics Committee (Pro00106957) on 5 January 2021. Participants can assess information leaflets at the beginning of the online survey, notifying them that consent was implied upon completion and submission of the online survey. Only data from individuals 18 years and older are reported in this study. Informed consent was obtained from all subjects.

### 3.3. Data Collection and Outcome Measures

MoreGoodDays is a self-subscription innovative text messaging program that delivers unidirectional computer-programmed messages to subscribers’ cell phones at 9 am Alberta Time each day for a year. Subscribers are requested to complete voluntary web-based program evaluation surveys on subscription (baseline) and then at six weeks, three months and six months after receiving the supportive text messages. Receiving supportive text messages by MoreGoodDays subscribers was not dependent on the survey’s completion. Participants were free to unsubscribe from the program at any time by texting back the word “STOP”. The survey usually takes 5 to 10 min to complete, and subscribers were not incentivized to encourage survey completion. The survey included a combination of sociodemographic and validated self-reported clinical questions [3]. Likely GAD was assessed using the Generalized Anxiety Disorder-7 (GAD-7) scale (GAD-7 scores ≥ 10 indicate likely GAD) [28]. Likely MDD was assessed using the Patient Health Questionnaire-9 (PHQ-9) scale (PHQ-9 score ≥ 10 indicates likely MDD). Similarly, likely PTSD and resilience level was assessed using the PTSD Checklist Civilian (PCL-C) (PCL-C score of 44 or more for likely PTSD) [29] and the Brief Resilience Scale (BRS) (a mean score ≤ 2.99 on the BRS indicates low resilience) [30]. For the severity estimates, we used the total scores for PCL-C (responses range from 17 to 85, and higher scores denote the poorer condition) and BRS (6 to 30, and lower scores denote the poorer condition). 

With respect to psychometric properties of the self-rated scales, the following Cronbach’s alpha has been reported: GAD-7 scale (0.77 for university students) [31], PHQ-9 (0.89 for the general public) [32], PCL-C (>0.70 for adolescents) [33] and BRS (0.78 for health workers) [34].

## 4. Satisfaction, Receptivity and Perception Outcome Measures

A primary outcome for this study was participants’ overall satisfaction with the MoreGoodDays program, evaluated using subscribers’ responses to the following question: “Using any number from 0 (not at all satisfied) to 10 (very satisfied), how would you rate your overall satisfaction MoreGoodDays program?”. Another primary outcome of this study was the changes in the mean scores for the PHQ-9, GAD-7 and PCL-C scales and the BRS from baseline to the three follow-up time points. The secondary outcome measures comprised changes in the prevalence of likely MDD, likely GAD, likely PTSD and low resilience from baseline to the three follow-up time points, as well as subscriber perceptions of MoreGoodDays program messages and receptivity to other technologically based services at six weeks, three months and six months from enrollment. Likert scale satisfaction responses to different aspects of the MoreGoodDays program were summarized as frequency counts of response categories and percentages. For analysis purposes, responses were collapsed into three points: agree or strongly agree, neutral and disagree or strongly disagree. Participants’ receptivity and perception of the MoreGoodDays program were assessed using a questionnaire evaluated on a 5-point Likert scale: often, sometimes, always, rarely and never. Likert scale satisfaction responses to various aspects of the MoreGoodDays program and participants’ anticipated receptivity to technology-based interventions (web-based counseling, video consultations for physical and mental health, telephone counseling, text and email messaging counseling, telephone consultations for physical and mental health) were also summarized as frequency counts of response categories and percentages. Although the satisfaction scale is not validated, these survey questionaries have previously been utilized in other studies to evaluate user satisfaction with similar supportive text messaging programs [21]. 

### Data Analysis

Data were analyzed with SPSS (version 25; IBM Corp, Armonk, NY, USA) statistical software for Windows [35], using descriptive and inferential analysis. Specifically, Chi-square/Fisher’s exact tests were used to assess differences in subscriber perceptions and satisfaction across the three follow-up time points. The McNemar Test was also used to examine the changes in the prevalence of likely MDD, likely GAD, likely PTSD and low resilience from baseline to the six-week follow-up time point. An analysis of variance was also used to examine the differences in mean scores for the PHQ-9, GAD-7 and PCL-C scales and the BRS from baseline to the three follow-up time points and from one follow-up time point to the other. Missing data were not imputed, and the results were based on completed survey responses.

## 5. Results

**Figure 1:**
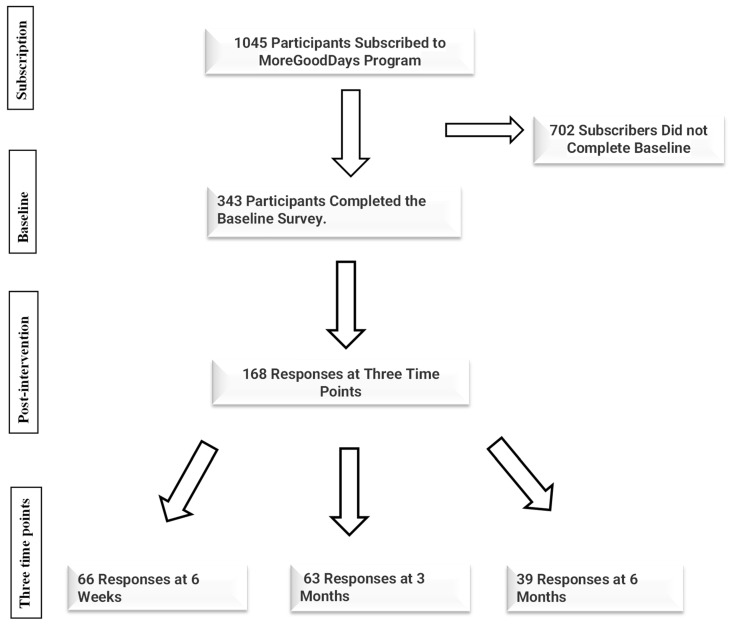
Study flowchart.

Overall, 343 MoreGoodDays participants completed the baseline survey, whilst 168 survey responses were received across the three time points (six weeks, three months and six months), as shown in Figure 1. 

The response rate for the baseline survey was 32.28%; however, because it was not possible to accurately estimate how many subscribers had unsubscribed from the MoreGoodDays program before reaching the various follow-up time points, the response rate for each of these time points could not be determined. The demographic characteristics of the respondents of MoreGoodDays can be assessed from a previously published manuscript [3]. In summary, most respondents to the baseline survey were females (271, 79.0%), white (250, 73.1%), employed (163, 47.4%) and single (170, 49.4%), lived with family and friends (148, 43.0%) and were not on any psychotropic medication (214, 62.2%). More than a third of the respondents had a mental health history of depression (124, 36.0%) and anxiety (135, 39.2%) [3].

### 5.1. Perceived Impact of MoreGoodDays Program

The total number of survey responses received at the three time points (six weeks, three months and six months) was 168. Table 1 gives an overview of the participants’ perceived impact of the MoreGoodDays daily messages received post-intervention at all three time points. Overall, 116 (69.1%) respondents agreed or strongly agreed that the MoreGoodDays text messages helped subscribers cope with stress, 108 (64.3%) reported the messages helped them cope with anxiety, 112 (66.7%) reported the messages helped them cope with depression, and 118 (70.3%) reported the messages helped them cope with loneliness. Similarly, 130 (77.3%) responses agreed or strongly agreed the MoreGoodDays messages made them feel connected to a support system, and 135 (80.4%) indicated it helped improve their overall mental well-being. In addition, 127 (75.6%) responses indicated subscribers felt hopeful about managing concerns with their mental health and 56 (35.2%) their substance use. Furthermore, 122 (72.6%) responses agreed or strongly agreed that MoreGoodDays text messages enhanced their quality of life. A total of 82% of responses agreed or strongly agreed that the MoreGoodDays program helped subscribers feel hopeful about managing mental health concerns and connected to a support system. At six months, 89.7% and 84.6% of responses agreed or strongly agreed that the program helped participants improve their overall mental well-being and quality of life, respectively, which was higher than the proportion of respondents who agreed or strongly agreed with these statements at six weeks (74.3% and 65.2%, respectively) and at three months (80.9% and 73.0%, respectively), although these differences did not achieve statistical significance. In addition, none of the other satisfaction measures for MoreGoodDays at the different time points achieved statistical significance.

### 5.2. Satisfaction and Feedback about MoreGoodDays Messages

Table 2 shows participants’ satisfaction and feedback about the MoreGoodDays program messages. The MoreGoodDays program was rated by participants for their overall satisfaction on a scale of 0–10, with zero indicating not at all satisfied and ten representing very satisfied. The mean satisfaction with the MoreGoodDays program messages was 8.74 (SD = 1.4). The majority of the responses, 154 (92.2%), indicated subscribers were very satisfied or satisfied with the frequency of the MoreGoodDays text messages, and the majority (127, 76.0%) specified that they preferred to receive the supportive text messages once daily. Overall, 164 (97.6%) MoreGoodDays responses for all three time points reported the text messages were always or often positive, 143 (89.9%) indicated the messages were always or often empowering, 142 (89.3%) said they were always or often succinct, and 139 (82.8%) reported the messages were always or often relevant. The majority of respondents (163, 97.6%) agreed they always or often read the MoreGoodDays text messages, 121 (72.5%) agreed they read the text messages and took time to reflect on the messages, and 25 (15.0%) read the text and took a positive or beneficial action. Furthermore, the majority (70, 41.9%) of responses indicated that subscribers sometimes returned to read MoreGoodDays text messages more than once. However, none of the other satisfaction measures for MoreGoodDays at the different time points achieved statistical significance.

Table 3 below shows MoreGoodDays subscribers’ projected receptivity to using different technology-based services as part of their health care. The results suggest that 132 (81%) survey responses agreed or strongly agreed with receiving web-based counseling and support, 121 (74.2%) with web-based addiction counseling and support and 132 (81%) and 136 (83.4%) telephone mental health counseling and text message mental health counseling, respectively, as part of their health care. With regards to addiction and mental health counseling, more than 70% agreed or strongly agreed to web-based addiction, telephone or text-message-based addiction counseling and support services, whilst only about half of responses indicated the willingness of subscribers to use email messaging for mental health counseling and support services. Again, about 142 (87.1%) of respondents agreed or strongly agreed with receiving consultations via video and telephone for mental health care and 136 (83.4%) for physical health care. Finally, most of the respondents, 132 (83.0%) MoreGoodDays respondents, indicated they preferred to receive the daily messages at 9 am in the morning, and 124 (78.0%) reported they preferred to receive the text messages for 12 months. 

Table 4 below shows the impact of the MoreGoodDays program on clinical outcomes over the three time points.

From Table 4, all the scales, GAD-7, BRS, PHQ 9 and PCLC, showed no significant differences in mean scores recorded at baseline, six weeks, three months and six months, indicating the program failed to achieve clinically significant positive or negative effects on subscribers’ self-reported psychological measures.

Table 5 presents the results of the changes in prevalence of likely GAD, likely MDD, likely PTSD and low resilience at baseline and at six weeks. 

Table 5 suggests there was no statistically significant difference in the prevalence of likely GAD, likely MDD, likely PTSD and low resilience at baseline and at six weeks. 

## 6. Discussion

The current study aimed to explore subscribers’ perspectives and satisfaction with the MoreGoodDays supportive text messaging program and the impact of the program on self-rated clinical measures during the COVID-19 pandemic. Our results show 8.74/10 overall satisfaction with the MoreGoodDays program, affirming the acceptability of text messaging support among the youth. Our study is corroborated by a study among university students on the acceptability and feasibility of receiving intervention by SMS text messaging, which reported that most of the participants were satisfied with the messages and would recommend the intervention to other students [36].

### 6.1. Perceived Impact of the MoreGoodDays Program

Overall, our results indicate that 77.3% of respondents agreed or strongly agreed the MoreGoodDays messages made participants feel connected to a support system, which is consistent with what was reported for the Text4Mood, Text4Hope, Text4Hope-addition and Text4PTSI programs [26,37,38]. Connection to a support system was particularly important for young adults during the pandemic stages when face-to-face contact was restricted. Our result also corroborates the Text4support satisfaction study among the patients receiving formal mental health and addiction services, in which 75.2% of the respondents indicated they felt connected to a support system from receiving the daily supportive text messages [21]. 

Across the three time points, our results show that a slightly higher proportion of responses (80.4%) indicated MoreGoodDays messages helped subscribers improve their overall mental well-being in comparison with the Text4Hope satisfaction study (75.6%) [26]. On the contrary, a slightly higher percentage of respondents in the Text4Mood and Text4PTSI evaluation study indicated that the text message intervention helped them improve their overall mental well-being (83.1% and 84%, respectively) [21,38]. Finally, in the sixth month post-intervention, 89.7% of respondents reported the program helped them improve their overall mental well-being. This may be a testament that the receipt of the messages for a longer duration may incur more perceived benefits. However, this should be interpreted with caution as it is more likely for those who need these messages to be retained for a longer program duration without opting out. 

Mental health issues lead to emotional imbalance, may increase the rate of suicide and interfere with interpersonal relationships, social interaction and academic achievement among adolescents and young adults [8,39]. Overall, 75.6% of respondents indicated they felt hopeful about managing concerns with their mental health, which is comparable to 76.7% of participants in the Text4Mood program who felt in charge of managing depression and anxiety [21]. 

One study [40] noted that the use of substances is not only detrimental to adolescents and youth but also imposes a huge burden on families and communities. This warrants a range of evidence-based treatment modalities, including usage of medication and school- and parent-based interventions. Unfortunately, only 32.5% of our respondents felt hopeful about managing their substance use concerns after receiving the MoreGoodDays messages. This is lower than the over 40% of respondents in the Text4Hope-Addiction and Text4PTSI program evaluation surveys who indicated they felt hopeful about managing concerns with their mental health or substance use after receiving the daily supportive text messages [37,38]. Unlike the MoreGoodDays messages, the messages in the Text4Hope-Addiction were more targeted at addiction support. This may offer explanations for the lower respondents in our study feeling hopeful about managing their substance use concerns.

### 6.2. Subscribers’ Perception of the MoreGoodDays Messages

Chronic stress can lead to the development of anxiety and depression, which can affect young adults’ everyday function and emotional balance [9,41]. A recent cross-sectional study among adolescents and young adults reported a relatively higher prevalence of both likely MDD and GAD, which can lead to increased levels of psychosocial dysfunction and poor academic performance [3,7]. More than 64% of the respondents agreed or strongly agreed that supportive text messages helped them cope with stress, anxiety and depression. This result is encouraging as young adults have been reported to be well-vested and adapted to mobile technology, especially text messaging, as a mode of communication [15]. This presents a fantastic opportunity for an innovative way to support their mental health [24]. Thus, as was evident in the Text4Hope program results for young adults [22], if the MoreGoodDays text messages were also clinically effective in reducing stress, anxiety and depression symptoms, the program can be used as an adjunct or complementary intervention for these groups. In addition, individuals interested in mobile phone programs have cited their convenience and ability to counteract isolation as reasons for their usage [42]. 

Transition to adulthood can be associated with physiological, cognitive, emotional, social and psychological changes that may result in loneliness [43]. Over 70.3% of respondents agreed or strongly agreed that the messages helped them cope with loneliness, and 80.4% reported it helped improve their mental well-being. Our results reemphasize the loneliness association with the transitional stage of this group and acknowledge the significance of the text message intervention in filling in the gap and helping subscribers cope with loneliness. Comparative to other studies, Text4Hope and Text4PTSI had lower numbers of respondents reporting the messages helped them cope with loneliness [26,38]. 

Approximately 72.0% of respondents in Text4Mood agreed or strongly agreed that the MoreGoodDays messages helped enhance their quality of life [21]. A slightly lower percentage, 61.7% of Text4Hope program respondents, agreed or strongly agreed that the messages helped enhance their quality of life [26] compared to 77% of respondents in the Text4PTSI program [38]. Consistent with the findings from other studies, the majority of MoreGoodDays subscribers reported the messages were always positive, empowering and succinct at all three time points [21,38]. More than 89.9% indicated the messages were always or often empowering and succinct, which is slightly more than what was observed in other studies. As previously alluded to, MoreGoodDays messages were crafted by the young adults for their peers. Notwithstanding, about 82.8% of participants reported the messages were always or often relevant, and 44.0% stated they were always relevant. On the contrary, in another study where CBT-based messages crafted by mental health professionals were used, twenty percent more respondents (61%) reported the messages were always relevant [26]. 

### 6.3. Delivery Preference of MoreGoodDays Messages

Overall, about 92% of respondents indicated they were very satisfied or satisfied with the frequency of text messages received, which is consistent with other studies [21,38]. Majority of MoreGoodDays respondents (76%) preferred to receive supportive text messages once daily, an increase of 8.0% in comparison to the Text4Mood study (68%) [21]. The Text4Mood program also reported that 12% of respondents preferred to receive supportive text messages once every other day compared to only 7.8% in our current study. MoreGoodDays subscribers’ (83%) preference was to receive messages at 9 am and for one year (78.0%), which may denote the importance young adults place on receiving the message to start their day as well as the value they place on the messages to help their day-to-day activities. MoreGoodDays subscribers may share the same sentiments as Text4Mood subscribers, who stated that the “text messages set a positive tone for their morning” [21].

### 6.4. Acceptance of Technology-Based Interventions

There is increasing acceptance of technology such as web- and text-based services among the general public, and many individuals perceive such services as supportive and timely with high levels of adherence [42,44]. MoreGoodDays subscribers welcomed all technology-based services as part of their health care. About 81% of the respondents agreed or strongly agreed with receiving web-based counseling and support. The most favorable eHealth technology among adolescents and young adults was receiving consultations via video (87.1%) and telephone for mental health care (83.4%), with respondents agreeing or strongly agreeing to receive consultation through this mode. Telephone mental health counseling (81%) and text message mental health counseling (83%) were also popular. This result adds to the growing body of literature that supports the inclusion of eHealth technology in mental healthcare delivery for young adults to reduce stigma-related issues associated with accessing face-to-face mental healthcare and enhance confidentiality [14,45]. Stigmatization has been linked to concealment, which can pose a barrier and may prevent young adults from accessing mental health care [45]. 

Moreover, delivery of CBT through mobile phones has been associated with significant positive outcomes for individuals with depression with sustained effect at three-month follow-up [46]. Contrary to the popularity of technology among the young adults in our study, participants in another study expressed different views about eHealth intervention, with only 25% of young people being positive about Internet-based health care [47]. However, agreeably, most young adults at least endorse the use of text messages [48].

Mental health support through email messaging seems to be comparatively unpopular. Only 51.2% of respondents indicated a willingness to use email messaging for counseling and support services, and only 47.2% were willing to use email messaging for addiction counseling and support services. Feedback from a study among university students after the receipt of eHealth intervention reported that SMS as a mode of delivery seems to be advantageous over email regarding when a message is read and the proportion of messages read [36]. Moreover, a lesser percentage of the participants in the email group reported reading all or almost all the messages compared to those in the SMS group. Another study using eHealth intervention also corroborates our results, as respondents accepted and preferred text messages over email and other web-based methods [48].

### 6.5. Clinical Outcomes

A systematic review reported that mental health apps have the ability to be effective, improve treatment accessibility and significantly reduce depression, stress and substance use [49]. The post hoc analysis in our study showed no significant differences in mean scores recorded at baseline to six weeks, three months and six months for any of the four clinical scales. In addition, there was no statistically significant difference in the prevalence of likely GAD, likely MDD, likely PTSD and low resilience at baseline and at six weeks. These suggested that although the MoreGoodDays messages had a high participant satisfaction rate, they did not make a significant clinical impact on subscribers with regard to anxiety, depression, PTSD and resilience symptoms. In contrast, the Text4Hope program outcomes for young adults showed a significant reduction in the mean scores on the GAD-7, PSS-10 and Composite Mental Health scores after six weeks of program participation, with the largest reduction being in mean scores for the GAD-7 scale (18.4%), with a small effect size overall [22]. In addition, there was also a significantly lower prevalence for likely MDD (25.2%) and suicidal thoughts/thoughts of self-harm (48.4%) in young adult subscribers of Text4Hope who had received the daily messages for six weeks compared with new subscribers [22]. It is possible the MoreGoodDays program did not achieve a significant reduction in any of the psychological measures because the daily messages were written exclusively by young adults and only for safety by the clinical team and thus did not fully align with CBT principles in comparison with the Text4Hope program messages which were written by mental health professionals based on CBT principles. The efficacy of CBT-based supportive text messages crafted by mental health professionals has also been observed in Text4Mood, Text4support, Text4Hope-Addiction and Text4PTSI programs [21,37,50,51]. CBT interventions have shown high efficacy in combination with medication or alone to combat anxiety, depression and other mental health morbidities [52].

### 6.6. Study Limitations

This study has some limitations worth mentioning. The study had a low response rate which is not uncommon for web-based survey completion rates which usually yield lower response rates and face challenges retaining participants for follow-up assessment compared to paper-based surveys [53]. This may have a negative effect on the study outcome and hence prevent its generalization. The sample size of participants who completed all follow-up assessments was small, which may lead to selection bias. Furthermore, the questionnaire used to measure user satisfaction with the MoreGoodDays program, although previously used in evaluating related programs, is nonetheless not a validated instrument. In addition, the self-rated scales used in this study, although standardized, are nonetheless not diagnostic. Also, there was no randomization in the MoreGoodDays sample; hence, there was no control group to compare the MoreGoodDays subscribers and non-subscribers in relation to the clinical impacts of the program. Therefore, in the absence of a control group, it remains unclear if this study cohort would have done worse on the measured clinical scale if they had not used the MoreGoodDays program, given the toll of the COVID-19 pandemic.

Finally, the MoreGoodDays program did not achieve clinical effectiveness, despite the high level of user satisfaction. This necessitates a review of the program messages which were all written by young adults, in particular, as comparable ResilienceNHope programs achieved clinical effectiveness. Notwithstanding these limitations, our study findings suggest that text-based health interventions have high satisfaction rates and may be valuable for bridging the psychological treatment gap for young adults if clinically effective messages are used for such programs.

## 7. Conclusions

Overall, the current study findings indicate that despite the lack of clinical effectiveness, the MoreGoodDays program was well perceived, and participants reported high satisfaction with the program messages, suggesting that e-communication is a suitable and acceptable mode of communication among young adults. Although this study achieved the accessibility, feasibility and reachability domains characteristic of other ResilienceNHope programs among young adults, the program did not achieve the clinical goals that have been achieved by other related programs. The lack of clinical effectiveness suggests that the MoreGoodDays messages need to be reviewed and enhanced by including cognitive-behavioral-therapy-based messages written by mental health clinicians, consistent with messages used in other ResilienceNHope programs such as Text4Hope and Text4PTSI, to improve the clinical effectiveness of the program.

The acceptance of technology-based interventions, coupled with the high subscriber satisfaction of the MoreGoodDays program and the clinical effectiveness recorded in related text-based programs, creates the possibility that an enhanced version of the MoreGoodDays program that incorporates CBT-based messages written by mental health professionals can be an add-on aid in future service planning by health service managers, policymakers and governments to improve the mental well-being of this cohort.

## Figures and Tables

**Table 1:** Perceived impact of daily messages post-intervention at all three time points.

Perceived Impact of MoreGoodDays	Six Weeks, n (%) N = 66	Three Months, n (%) N = 63	Six Months, n (%) N = 39	Total, n (Average %) N= 168	Chi²/Fisher Exact Value	*p*-Value
**Helped participants to cope with stress**						
Agree/Strongly agree	40 (60.7)	47 (74.6)	29 (74.4)	116 (69.1)	* 5.35	0.76
Neutral	23 (34.8)	14 (22.2)	9 (23.1)	46 (27.4)		
Disagree/Strongly disagree	3 (4.5)	2 (3.2)	1 (2.6)	6 (3.6)		
**Helped participants to cope with anxiety**						
Agree/Strongly agree	39 (59.1)	40 (63.5)	29 (74.3)	108 (64.3)	* 8.19	0.38
Neutral	25 (37.9)	19 (30.2)	9 (23.1)	53 (31.5)		
Disagree/Strongly disagree	2 (3.0)	4 (6.3)	1 (2.6)	7 (4.2)		
**Helped participants to cope with depression**						
Agree/Strongly agree	37 (57.6)	46 (73.1)	28 (71.8)	112 (66.7)	* 6.46	0.59
Neutral	21 (31.8)	13 (20.6)	10 (25.6)	44 (26.2)		
Disagree/Strongly disagree	7 (10.6)	4(6.3)	1 (2.6)	12 (7.2)		
**Helped participants to cope with loneliness**						
Agree/Strongly agree	45 (68.2)	45 (71.4)	28 (71.8)	118 (70.3)	* 3.22	0.92
Neutral	15 (22.7)	13 (20.6)	9 (23.1)	37 (22.0)		
Disagree/Strongly disagree	6 (9.1)	5 (7.9)	2 (5.1)	13 (7.7)		
**Participants felt connected to a support system**						
Agree/Strongly agree	50 (75.8)	48 (76.2)	32 (82.1)	130 (77.3)	* 7.33	0.28
Neutral	9 (13.6)	14 (22.2)	6 (15.4)	29 (17.3)		
Disagree/Strongly disagree	7(10.6)	1 (1.6)	1 (2.6)	9 (5.4)		
**Helped participants feel hopeful about managing mental health concerns**						
Agree/Strongly agree	50 (75.8)	45 (71.4)	32 (82.0)	127 (75.6)	* 6.05	0.64
Neutral	12 (18.2)	15 (23.8)	5 (12.8)	32 (19)		
Disagree/Strongly disagree	4 (6.0)	3 (4.8)	2 (5.2)	9 (5.4)		
**Helped participants feel hopeful about managing substance use concerns**						
Agree/Strongly agree	0 (32.4)	23 (39.0)	13 (33.3)	56 (35.2)	* 5.86	0.67
Neutral	32 (52.5)	30 (50.8)	21 (53.8)	83 (52.2)		
Disagree/Strongly disagree	9 (14.8)	6 (10.2)	5 (12.8)	20 (12.5)		
**Helped participants improve their overall mental well-being**						
Agree/Strongly agree	49 (74.3)	51 (80.9)	35 (89.7)	135 (80.4)	* 7.07	0.50
Neutral	13 (19.7)	10 (15.9)	4 (10.3)	27 (16.1)		
Disagree/Strongly disagree	4 (6.0)	2 (3.2)	0 (0.0)	6 (3.6)		
**Enhanced their quality of life**						
Agree/Strongly agree	43 (65.2)	46 (73.0)	33 (84.6)	122 (72.6)	* 7.57	0.44
Neutral	19 (28.8)	14 (22.2)	6 (15.4)	39 (23.2)		
Disagree/Strongly disagree	4 (6.0)	3 (4.8)	0 (0.0)	7 (4.2)		

* Fisher Exact Value.

**Table 2:** Participants’ feedback at three points after the intervention.

Feedback	Six Weeks n (%) N = 66	Three Months n (%) N = 63	Six Months n (%) N = 39	Total n (%) N = 168	Chi² Value	*p*-Value
**The MoreGoodDays text messages were positive.**						
Always	49 (74.2)	49 (77.8)	28 (71.8)	126 (75)	* 2.92	0.93
Often	15 (22.7)	13 (20.6	10 (25.6)	38 (22.6)		
Sometimes	1 (1.5)	0 (0.0)	1 (2.6)	2 (1.2)		
Rarely	1 (1.5)	1 (1.6)	0 (0.0)	2 (1.2)		
**The MoreGoodDays text messages were empowering.**						
Always	31 (50.8)	34 (57.6)	21 (53.8)	86 (54.1)	* 4.53	0.61
Often	21 (34.4)	20 (33.9)	16 (41.0)	57 (35.8)		
Sometimes	6 (9.8)	5 (8.5)	2 (5.1)	13 (8.2)		
Rarely	3 (4.9)	0 (0.0)	0 (0.0)	3 (1.9)		
**The MoreGoodDays text messages were succinct.**						
Always	36 (59.0)	34 (57.6)	21 (53.8)	91 (57.2)	* 4.53	0.94
Often	19 (31.1)	19 (32.2)	13 (33.3)	51 (32.1)		
Sometimes	5 (8.2)	6 (10.2)	4 (10.3)	15 (9.4)		
Rarely	1 (1.6)	0 (0.0)	0 (0.0)	1 (0.6)		
Never	0 (0.0)	0 (0.0)	1 (2.6)	1 (0.6)		
**The MoreGoodDays text messages were relevant.**						
Always	26 (39.4)	30 (47.6)	17 (43.6)	73 (43.5)	* 4.96	0.82
Often	28 (42.4)	24 (38.1)	14 (35.9)	66 (39.3)		
Sometimes	10 (15.2)	8 (12.7)	7 (17.9)	25 (14.9)		
Rarely	2(3.0)	0 (0.0)	1 (2.6)	3 (1.8)		
Never	0 (0.0)	1 (1.6)	0 (0.0)	1 (0.6)		
**Frequency reading the MoreGoodDays text messages.**						
Always	55 (84.6)	53 (84.1)	34 (87.2)	142 (85)	* 0.92	0.97
Often	9 (13.8)	8 (12.7)	4(10.3)	21 (12.6)		
Sometimes	1 (1.5)	2 (3.2)	1 (2.6)	4 (2.4)		
**Action taken after reading text messages.**						
Read the text and took no action.	11 (16.9)	7 (11.1)	3 (7.7)	21 (12.6)	* 2.96	0.58
Read the text and took time to reflect on the message.	45 (69.2)	48 (76.2)	28 (71.8)	121 (72.5)		
Read the text and took a positive or beneficial action.	9 (13.8)	8 (12.7)	8 (20.5)	25 (15.0)		
**Return to read MoreGoodDays text messages more than once.**						
Always	7(10.8)	2 (3.2)	4 (10.3)	13 (7.8)	* 9.63	0.29
Often	11 (16.9)	11 (17.5)	9 (23.1)	31 (18.6)		
Sometimes	29 (44.6)	26 (41.3)	15 (38.5)	70 (41.9)		
Rarely	10 (15.4)	19 (30.2)	10 (25.6)	39 (23.4)		
Never	8 (12.3)	5 (7.9)	1 (2.6)	14 (8.4)		
**Satisfaction with the frequency of the MoreGoodDays text messages.**						
Very satisfied/Satisfied	60 (92.3)	60 (95.2)	34 (87.1)	154 (92.2)	* 4.98	0.54
Neutral	5 (7.7)	3 (4.8)	4 (10.3)	12 (7.2)		
Dissatisfied	0 (0.0)	0 (0.0)	1 (2.6)	1 (0.6)		
**Frequency participants prefer to receive supportive text messages.**						
Twice daily	9 (13.8)	13 (20.6)	4 (10.3)	26 (15.6)	* 5.70	0.43
Once daily	50 (76.9)	47 (74.6)	30 (76.9)	127 (76.0)		
Once every other day	6 (9.2)	3 (4.8)	4 (10.3)	13 (7.8)		
Once weekly	0 (0.0)	0 (0.0)	1 (2.6)	1 (0.6)		
**Participants’ overall satisfaction with MoreGoodDays? 0 (not at all satisfied) to 10 (very satisfied)**						
**0–3**	0 (0.0)	0 (0.0)	0 (0.0)	0 (0.0)	* 6.99	0.90
**4**	1(1.5%)	0 (0.0)	0 (0.0)	1 (0.6)		
**5**	2 (3.1)	1 (1.6)	2 (5.1)	5 (3.0)		
**6**	5 (7.7)	2 (3.2)	2 (5.1)	9 (5.4)		
**7**	6 (9.2)	5 (7.9)	1 (2.6)	12 (7.2)		
**8**	13 (20.0)	12 (19.0)	8 (20.5)	33 (19.8)		
**9**	15 (23.1)	18 (28.6)	8 (20.5)	41 (24.6)		
**10 (Very satisfied)**	23 (35.4)	25 (39.7)	18 (46.2)	66 (39.5)		

* Chi^2^ Value.

**Table 3:** Participants’ feedback on willingness to use other innovative web-based mental health applications.

Perceived Impact of MoreGoodDays	Six Weeks, n (%) N = 63	Three Months, n (%) N = 62	Six Months, n (%) N = 38	Total, n (Average %) N = 163
**Web-based mental health counseling and support.**				
Agree/Strongly agree	51(81)	51 (83.9)	29 (76.3)	132 (81)
Neutral	8 (12.7)	5 (8.1)	7 (18.4)	20 (12.3)
Disagree/Strongly disagree	4 (6.4)	5(8.1)	2 (5.2)	11 (6.7)
**Web-based addiction counseling and support.**				
Agree/Strongly agree	46 (73)	48 (77.4)	27 (71.1)	121 (74.2)
Neutral	13 (20.6)	9 (14.5)	9 (23.7)	31 (19.0)
Disagree/Strongly disagree	4 (6.4)	5 (8.1)	2 (5.3)	11 (6.7)
**Telephone mental health counseling and support.**				
Agree/Strongly agree	49 (77)	49 (79)	34 (89.5)	132 (81)
Neutral	8 (12.7)	7 (11.3)	3 (7.9)	18 (11)
Disagree/Strongly disagree	6 (9.5)	6 (9.7)	1 (2.6)	13 (7.9)
**Telephone addiction counseling and support.**				
Agree/Strongly agree	45 (71.4)	46 (74.2)	31 (81.5)	122 (74.8)
Neutral	15 (23.8)	10 (16.1)	5 (13.2)	30 (18.4)
Disagree/Strongly disagree	3 (4.8)	6 (9.7)	2 (5.3)	11 (6.7)
**Text messaging for mental health counseling and support.**				
Agree/Strongly agree	54 (85.7)	51 (82.2)	31(81.6)	136 (83.4)
Neutral	6 (9.5)	6 (9.7)	6 (15.8)	18 (11)
Disagree/Strongly disagree	3 (4.8)	5 (8.1)	1 (2.6)	9 (5.5)
**Text messaging for addiction counseling and support.**				
Agree/Strongly agree	46 (73)	48 (77.4)	26 (68.4)	120 (73.6)
Neutral	12 (19.0)	9 (14.5)	9 (23.7)	30 (18.4)
Disagree/Strongly disagree	5 (7.9)	5 (8.1)	3 (7.9)	13 (8.0)
**Email messaging for mental health counseling and support**				
Agree/Strongly agree	31 (50)	31 (50)	21 (55.2)	83 (51.2)
Neutral	21 (33.9)	17 (27.4)	9 (23.7)	47 (29.0)
Disagree/Strongly disagree	10 (16.1)	14 (22.6)	8 (21.1)	32 (19.7)
**Email messaging addiction counseling and support.**				
Agree/Strongly agree	27 (42.9)	29 (46.8)	21 (55.3)	77 (47.2)
Neutral	24 (38.1)	19 (30.6)	9 (23.7)	52 (31.9)
Disagree/Strongly disagree	12 (19.1)	14 (22.6)	8 (21.1)	34 (20.8)
**Consultation via video conferencing for mental health care.**				
Agree/Strongly agree	52 (82.5)	55 (88.7)	35 (92.1)	142 (87.1)
Neutral	8 (12.7)	4 (6.5)	2 (5.3)	14 (8.6)
Disagree/Strongly disagree	3 (4.8)	3 (4.8)	1 (2.6)	7 (4.3)
**Consultation via video conferencing for physical health care.**				
Agree/Strongly agree	50 (79.3)	55 (88.7)	34 (89.5)	139 (85.3)
Neutral	10 (15.9)	5 (8.1)	1 (2.6)	16 (9.8)
Disagree/Strongly disagree	3 (4.8)	2 (3.2)	3 (7.9)	8 (4.9)
**Consultation via telephone conferencing for mental health care.**				
Agree/Strongly agree	49 (77.8)	52 (83.9)	35 (92.1)	136 (83.4)
Neutral	8 (12.7)	6 (9.7)	1 (2.6)	15 (9.2)
Disagree/Strongly disagree	6 (9.6)	4 (6.4)	2 (5.3)	12 (7.4)
**Consultation via telephone conferencing for physical health care.**				
Agree/Strongly agree	47 (74.6)	51 (82.2)	34 (89.4)	132 (81)
Neutral	11 (17.5)	7 (11.3)	2 (5.3)	20 (12.3)
Disagree/Strongly disagree	5 (8.0)	4 (6.4)	2 (5.2)	11 (6.8)
**MoreGoodDays text message delivery—Time preference**				
**What time of the day would you prefer to receive the daily messages?**				
Morning (9 am)	49 (77.8)	51 (87.9)	32 (84.2)	132 (83.0)
Noon (12 pm)	11 (17.5)	1 (1.7)	2 (5.3)	14 (8.8)
Afternoon (3 pm)	2 (3.2)	4 (6.9)	1 (2.6)	7 (4.4)
Evening (6 pm)	0 (0.0)	1 (1.7)	2 (5.3)	3 (1.9)
Night (9 pm)	1 (1.6)	1 (1.7)	1 (2.6)	3 (1.9)
**For how long would you prefer to receive the daily messages?**				
3 Months	9 (14.3)	2 (3.4)	2 (5.3)	13 (8.2)
6 Months	6 (9.5)	8 (13.8)	4 (10.5)	18 (11.3)
9 Months	2 (3.2)	2 (3.4)	0 (0.0)	4 (2.5)
12 Months	46 (73.0)	46 (79.3)	32 (84.2)	124 (78)

**Table 4:** One-way ANOVA showing the mean scores and Fand *p*-values for the PHQ-9, GAD-7 and PCL-C scales and the BRS from baseline to the three follow-up time points.

Scale	N	Mean	Std. Deviation	Std. Error	95% Confidence Interval for Mean		F-Value	Sig
					Lower Bound	Upper Bound		
**BRS Average**							0.79	0.50
Baseline	323	2.82	0.82	0.05	2.73	2.91		
6 Weeks	74	2.79	0.75	0.09	2.62	2.96		
3 Months	68	2.86	0.84	0.10	2.66	3.06		
6 Months	40	3.02	0.77	0.12	2.77	3.26		
Total	505	2.84	0.81	0.04	2.77	2.91		
**PHQ Total**							2.79	0.40
Baseline	314	12.31	7.36	0.42	11.49	13.13		
6 Weeks	70	10.57	6.63	0.79	8.99	12.15		
3 Months	65	10.92	7.29	0.90	9.12	12.73		
6 Months	39	9.52	6.34	1.01	7.46	11.57		
Total	488	11.65	7.21	0.33	11.01	12.29		
**GAD Total**							2.40	0.07
Baseline	311	10.21	6.02	0.34	9.54	10.88		
6 Weeks	68	8.93	6.14	0.74	7.44	10.14		
3 Months	64	8.82	5.85	0.73	7.53	10.27		
6 Months	39	8.23	5.43	0.87	6.47	9.99		
Total	482	9.68	9.68	0.27	9.15	10.22		
**PCLC Total**							1.80	0.15
Baseline	309	44.28	17.61	1.00	42.31	46.26		
6 Weeks	67	41.73	16.67	2.04	37.67	45.79		
3 Months	63	42.06	17.16	2.16	37.74	46.38		
6 Months	39	38.08	15.57	2.49	33.03	43.13		
Total	478	43.13	17.31	.79	41.57	44.68		

GAD—Generalized Anxiety Disorder; BRS—Brief Resilience Scale; PHQ—Patient Health Questionnaire; PTSD—Post-traumatic Stress Disorder; PCLC—Post-traumatic Stress Disorder Civilian Checklist; Std—Standard; Sig—Significance.

**Table 5:** Changes in the prevalence of likely GAD, likely MDD, likely PTSD and low resilience.

Clinical Condition	Prevalence		Change from Baseline	df	*p*-Value
	Baseline	Six Weeks			
Likely GAD	25 (51.0%)	24 (49.0%)	−2.0%	1	0.2
Likely MDD	29 (58.0%)	33 (66.0%)	+8.0%	1	1.0
Likely PTSD	24 (49.0%)	26 (53.1%)	+4.1%	1	0.8
Low resilience	34 (64.2%)	35 (66.0%)	+1.8%	1	1.0

df—Degree of Freedom.

## Data Availability

Data for this study is available on reasonable request from the corresponding author.

## References

[1] Fawzy M., Hamed S.A. (2017). Prevalence of psychological stress, depression and anxiety among medical students in Egypt. Psychiatry Res..

[2] Fergusson D.M., Boden J.M., Horwood L.J. (2007). Recurrence of major depression in adolescence and early adulthood, and later mental health, educational and economic outcomes. Br. J. Psychiatry.

[3] Agyapong B., Shalaby R., Hay K., Pattison R., Eboreime E., Korthuis M., Wei Y., Agyapong V.I.O. (2023). Exploring Sociodemographic Characteristics, Adverse Childhood Experience, and Mental Health History as Predictors of Anxiety and Depression among Adolescents and Young Adults: Findings from the MoreGoodDays Support Program in Alberta, Canada. Behav. Sci..

[4] Garber J., Weersing V.R. (2010). Comorbidity of Anxiety and Depression in Youth: Implications for Treatment and Prevention. Clin. Psychol..

[5] Costello E.J., Mustillo S., Erkanli A., Keeler G., Angold A. (2003). Prevalence and development of psychiatric disorders in childhood and adolescence. Arch. Gen. Psychiatry.

[6] Richardson L.P., Russo J.E., Lozano P., McCauley E., Katon W. (2010). Factors associated with detection and receipt of treatment for youth with depression and anxiety disorders. Acad. Pediatr..

[7] Aloufi M.A., Jarden R.J., Gerdtz M.F., Kapp S. (2021). Reducing stress, anxiety and depression in undergraduate nursing students: Systematic review. Nurse Educ. Today.

[8] Gould M.S., King R., Greenwald S., Fisher P., Schwab-Stone M., Kramer R., Flisher A.J., Goodman S., Canino G., Shaffer D. (1998). Psychopathology associated with suicidal ideation and attempts among children and adolescents. J. Am. Acad. Child. Adolesc. Psychiatry.

[9] Mangione C.M., Barry M.J., Nicholson W.K., Cabana M., Chelmow D., Coker T.R., Davidson K.W., Davis E.M., Donahue K.E., Jaén C.R. (2022). Screening for Depression and Suicide Risk in Children and Adolescents: US Preventive Services Task Force Recommendation Statement. JAMA.

[10] Ma Z., Zhao J., Li Y., Chen D., Wang T., Zhang Z., Chen Z., Yu Q., Jiang J., Fan F. (2020). Mental health problems and correlates among 746,217 college students during the coronavirus disease 2019 outbreak in China. Epidemiol. Psychiatr. Sci..

[11] Huang Y., Zhao N. (2020). Generalized anxiety disorder, depressive symptoms and sleep quality during COVID-19 outbreak in China: A web-based cross-sectional survey. Psychiatry Res..

[12] Nwachukwu I., Nkire N., Shalaby R., Hrabok M., Vuong W., Gusnowski A., Surood S., Urichuk L., Greenshaw A.J., Agyapong V.I.O. (2020). COVID-19 Pandemic: Age-Related Differences in Measures of Stress, Anxiety and Depression in Canada. Int. J. Environ. Res. Public Health.

[13] Liu C.H., Zhang E., Wong G.T.F., Hyun S., Hahm H.C. (2020). Factors associated with depression, anxiety, and PTSD symptomatology during the COVID-19 pandemic: Clinical implications for U.S. young adult mental health. Psychiatry Res..

[14] Ouellet-Morin I., Robitaille M.-P., Juster R.-P. (2021). Mobile Applications to Promote Youth Mental Health: Opportunities and Challenges. Sante Ment Que.

[15] Valkenburg P.M., Peter J. (2011). Online communication among adolescents: An integrated model of its attraction, opportunities, and risks. J. Adolesc. Health.

[16] Thoma N., Pilecki B., McKay D. (2015). Contemporary cognitive behavior therapy: A review of theory, history, and evidence. Psychodyn. Psychiatry.

[17] Agyapong B., Brett-MacLean P., Burback L., Agyapong V.I.O., Wei Y. (2023). Interventions to Reduce Stress and Burnout among Teachers: A Scoping Review. Int. J. Environ. Res. Public Health.

[18] Reid S.C., Kauer S.D., Hearps S.J., Crooke A.H., Khor A.S., Sanci L.A., Patton G.C. (2011). A mobile phone application for the assessment and management of youth mental health problems in primary care: A randomised controlled trial. BMC Fam. Pract..

[19] Danese A., Martsenkovskyi D. (2023). Editorial: Measuring and Buffering the Mental Health Impact of the War in Ukraine in Young People. J. Am. Acad. Child. Adolesc. Psychiatry.

[20] Sirotkin A.V., Pavlíková M., Hlad Ľ., Králik R., Zarnadze I., Zarnadze S., Petrikovičová L. (2023). Impact of COVID-19 on university activities: Comparison of experiences from Slovakia and Georgia. Sustainability.

[21] Agyapong V.I., Mrklas K., Juhás M., Omeje J., Ohinmaa A., Dursun S.M., Greenshaw A.J. (2016). Cross-sectional survey evaluating Text4Mood: Mobile health program to reduce psychological treatment gap in mental healthcare in Alberta through daily supportive text messages. BMC Psychiatry.

[22] Agyapong B., Shalaby R., Vuong W., Gusnowski A., Surood S., Greenshaw A.J., Wei Y., Agyapong V.I.O. (2023). Text4Hope Effectiveness in Reducing Psychological Symptoms among Young Adults in Canada: Longitudinal and Naturalistic Controlled Program Evaluation. J. Clin. Med..

[23] Kickstand. More Good Days. Edmonton. https://mykickstand.ca/online-care#more-good-days.

[24] Agyapong B., Shalaby R., Wei Y., Agyapong V.I. (2022). Can ResilienceNhope, an evidence-based text and email messaging innovative suite of program help to close the psychological treatment and mental health literacy gaps in college students?. Front. Public Health.

[25] ResilienceNHope Edmonton: ResilienceNHope. https://www.resiliencenhope.org/.

[26] Shalaby R., Vuong W., Hrabok M., Gusnowski A., Mrklas K., Li D., Snaterse M., Surood S., Cao B., Li X.-M. (2021). Gender Differences in Satisfaction With a Text Messaging Program (Text4Hope) and Anticipated Receptivity to Technology-Based Health Support During the COVID-19 Pandemic: Cross-sectional Survey Study. JMIR Mhealth Uhealth.

[27] Statistics Canada (2023). Census of Population.

[28] Spitzer R.L., Kroenke K., Williams J.B., Löwe B. (2006). A brief measure for assessing generalized anxiety disorder: The GAD-7. Arch. Intern. Med..

[29] Weathers F.W., Litz B.T., Keane T.M., Palmieri P.A., Marx B.P., Schnurr P.P. (2013). The PTSD Checklist for DSM-5 (PCL-5). Affair USDoV.

[30] Smith B.W., Dalen J., Wiggins K., Tooley E., Christopher P., Bernard J. (2008). The brief resilience scale: Assessing the ability to bounce back. Int. J. Behav. Med..

[31] Manzar D., Salahuddin M., Alghadir A., Anwer S., Peter S., Bahammam A.S., Pandi-Perumal S.R. (2021). Psychometric properties of the Generalized Anxiety Disorder-7 Scale in Ethiopian university students. Bull. Menn. Clin..

[32] Saldivia S., Aslan J., Cova F., Vicente B., Inostroza C., Rincón P. (2019). Psychometric characteristics of the Patient Health Questionnaire (PHQ-9). Rev. Med. Chil..

[33] Chen W., Gao R., Yang T. (2021). Factor Structure and Psychometric Properties for the PTSD Checklist of Chinese Adolescents in the Closed Period after the COVID-19 Outbreak. Int. J. Environ. Res. Public Health.

[34] Soer R., Dijkstra M.W.M.C.S., Bieleman H.J., Stewart R.E., Reneman M.F., Oosterveld F.G.J., Schreurs K.M.G. (2019). Measurement properties and implications of the Brief Resilience Scale in healthy workers. J. Occup. Health.

[35] IBM Support (2019). Release Notes—IBM® SPSS® Statistics 25.0.

[36] Bendtsen M., Bendtsen P. (2014). Feasibility and user perception of a fully automated push-based multiple-session alcohol intervention for university students: Randomized controlled trial. JMIR mHealth uHealth.

[37] Obuobi-Donkor G., Shalaby R., Vuong W., Agyapong B., Hrabok M., Gusnowski A., Surood S., Greenshaw A.J., Agyapong V.I. (2023). Effects of Text4Hope-Addiction Support Program on Cravings and Mental Health Symptoms: Results of a Longitudinal Cross-sectional Study. JMIR Form. Res..

[38] Obuobi-Donkor G., Eboreime E., Shalaby R., Agyapong B., Phung N., Eyben S., Wells K., Dias R.d.L., Hilario C., Jones C. (2023). User Satisfaction With a Daily Supportive Text Message Program (Text4PTSI) for Public Safety Personnel: Longitudinal Cross-Sectional Study. JMIR Form. Res..

[39] Rohde P., Clarke G.N., Lewinsohn P.M., Seeley J.R., Kaufman N.K. (2001). Impact of comorbidity on a cognitive-behavioral group treatment for adolescent depression. J. Am. Acad. Child. Adolesc. Psychiatry.

[40] Kulak J.A., Griswold K.S. (2019). Adolescent Substance Use and Misuse: Recognition and Management. Am. Fam. Physician.

[41] Patel D., Kas M.J., Chattarji S., Buwalda B. (2019). Rodent models of social stress and neuronal plasticity: Relevance to depressive-like disorders. Behav. Brain Res..

[42] Proudfoot J.G., Parker G.B., Pavlovic D.H., Manicavasagar V., Adler E., Whitton A.E. (2010). Community attitudes to the appropriation of mobile phones for monitoring and managing depression, anxiety, and stress. J. Med. Internet Res..

[43] Toney-Butler T.J., Siela D. (2023). StatPearls.

[44] Torous J., Staples P., Shanahan M., Lin C., Peck P., Keshavan M., Onnela J.-P. (2015). Utilizing a Personal Smartphone Custom App to Assess the Patient Health Questionnaire-9 (PHQ-9) Depressive Symptoms in Patients With Major Depressive Disorder. JMIR Ment. Health.

[45] Hankir A.K., Northall A., Zaman R. (2014). Stigma and mental health challenges in medical students. BMJ Case Rep..

[46] Watts S., Mackenzie A., Thomas C., Griskaitis A., Mewton L., Williams A., Andrews G. (2013). CBT for depression: A pilot RCT comparing mobile phone vs. computer. BMC Psychiatry.

[47] Wozney L., Baxter P., Newton A.S. (2015). Usability evaluation with mental health professionals and young people to develop an internet-based cognitive-behaviour therapy program for adolescents with anxiety disorders. BMC Pediatr..

[48] Moore S.C., Crompton K., van Goozen S., van den Bree M., Bunney J., Lydall E. (2013). A feasibility study of short message service text messaging as a surveillance tool for alcohol consumption and vehicle for interventions in university students. BMC Public Health.

[49] Donker T., Petrie K., Proudfoot J., Clarke J., Birch M.R., Christensen H. (2013). Smartphones for smarter delivery of mental health programs: A systematic review. J. Med. Internet Res..

[50] Noble J.M., Vuong W., Surood S., Urichuk L., Greenshaw A.J., Agyapong V.I.O. (2021). Text4Support Mobile-Based Programming for Individuals Accessing Addictions and Mental Health Services-Retroactive Program Analysis at Baseline, 12 Weeks, and 6 Months. Front. Psychiatry.

[51] Obuobi-Donkor G., Shalaby R., Eboreime E., Agyapong B., Phung N., Eyben S., Wells K., Hilario C., Dias R.d.L., Jones C. (2023). Text4PTSI: A Promising Supportive Text Messaging Program to Mitigate Psychological Symptoms in Public Safety Personnel. Int. J. Environ. Res. Public Health.

[52] Dardas L.A., Xu H., Franklin M.S., Scott J., Vance A., van de Water B., Pan W. (2023). Cognitive behavioural therapy and medication for treatment of adolescent depression: A network meta-analysis. Behav. Cogn. Psychother..

[53] Daikeler J., Bošnjak M., Lozar Manfreda K. (2020). Web versus other survey modes: An updated and extended meta-analysis comparing response rates. J. Surv. Stat. Methodol..

